# Explaining the monoaural directional hearing of the moth *Achroia grisella*

**DOI:** 10.1098/rsif.2024.0752

**Published:** 2025-01-15

**Authors:** Lara Díaz-García, Andrew Reid, James F. C. Windmill

**Affiliations:** ^1^ Centre for Ultrasonic Engineering, University of Strathclyde, Glasgow, UK

**Keywords:** bioacoustics, finite element modelling, insect hearing, Lepidoptera, Pyralidae, acoustic sensing

## Abstract

*Achroia grisella* (Fabricius, 1794) (Lepidoptera: Pyralidae) is a pyralid moth with two ears in its abdomen that it uses for detecting mates and predators. Despite no connection between the two ears having been found and no other elements having been observed through X-ray scans of the moth, it seems to be capable of directional hearing with just one ear when one of them is damaged. It is therefore suspected that the morphology of the eardrum can provide directional cues for sound localization. Here, we use finite element modelling software COMSOL to model a simplified version of the eardrum, an elliptical plate with two sections of different thicknesses and a mass load at the centre of the thin section, to try to determine if the morphology of the ear is responsible for the moth’s monoaural directional hearing. Results indicate that the resonance mode and directionality response of the elliptical plate with two thicknesses and a mass load match that of the moth closely and provide an enhanced response to sounds coming from the front of the moth. Damping is also considered in the resonant mode, and it is observed to improve the resemblance of the simulation to real moth ear measurements.

## 1. Background

Mechano-sensing is a useful tool that many animals use to interact with their environment. Hearing, which occurs through the use of mechano-sensory systems, allows animals to listen for conspecifics (be it mating or warning calls), potential predators, potential prey and many other phenomena around them [1,2]. A hearing sense, although ubiquitous within mammals, is not always present or alike among insects [3,4]. The Insecta class presents a well-known biodiversity [5]; their hearing organs have evolved separately between 15 and 20 times [6], and they are located all across their bodies and present various mechanisms of sound detection [7].

In addition to sound detection, pinpointing the source of a sound can also be desirable, an ability known as directionality [8,9]. Finding the location of prey based on the sounds they make, localizing a potential mate that is advertising its presence, or coming to the rescue of their offspring in response to a distress call all benefit from knowing where the sound is coming from and, therefore, from directional hearing. For a moth that has acoustically detected the presence of a dangerously close bat, it might be enough to initiate erratic flight in the hope of avoiding it; if what it is trying to do is find the precise location of a potential mate, then it is not enough to know that the mate is somewhere close by, it is also necessary to know exactly where they are calling from [8].

Directionality in larger animals, like mammals, is mostly achieved by comparison of the input of the two ears that sit symmetrically and far apart on the body. The brain will then compute the difference in time of arrival, intensity and phase at each ear and determine where the sound is coming from. The larger the distance between the ears relative to the wavelength of the sound, the greater the cues for determining direction of the sound will be [1]. When body size is reduced, as it generally is for insects, this tactic no longer works; if the eardrums sit too closely to represent a meaningful difference for the wavelength that is being listened to, the sound will essentially reach both ears simultaneously. The ability to measure small time differences of arrival is limited by the variation in the length of the refractory period (time of recovery) of the receptor neuron after firing an action potential, known as the spike timing jitter. Although the spike timing jitter of *Achroia grisella* is not known, other exemplary time jitters of sensory neurons are 76 µs for *Ormia ochracea* (Bigot, 1889) (Diptera: Tachinidae), a parasitic fly further discussed later [10]; or 1 µs in *Eigenmannia virescens* (Valenciennes, 1847) (Gymnotiformes: Sternopygidae), a fish which can sense electrical signals [11].

Insects have developed workarounds to achieve directional hearing, and due to the diversity in hearing organs, it is to be expected that different solutions have evolved. One of the most studied insects capable of directionality is *O. ochracea*, a species of parasitic fly that locates its host through interconnected tympana [12–14]. A well-known insect order capable of directional hearing is Orthoptera, which includes crickets and locusts, among others. In general terms, insects within Orthoptera achieve directional hearing due to a tracheal system that connects the inside of the tympana to the outside, providing more than one pathway for sound to reach the eardrum [7,15,16]. Additionally, sound intensity can also code interaural differences through effects on latency [17,18].

*Achroia grisella*, known as the lesser wax moth, is a nocturnal moth that infests beehives. This species is one of many moths with hearing in the ultrasonic range, which serves as a tool to escape one of their main predators, bats [19]. While defence against bats is the most common theory for the emergence of hearing in moths [4,20,21], it has lately been reported that three of the independent origins of hearing in moths and butterflies (collectively known as Lepidoptera) pre-date the appearance of echolocation in bats [22].

Intraspecific communication in moths, on the other hand, is much rarer [23], which is why the mating ritual of *A. grisella*, which involves the male signalling with ultrasonic pulse trains generated while fanning the wings, which the females listen to and track, is peculiar [24,25]. The lesser wax moth is known to be capable of directional hearing, but the mechanism for this is unlike any of the previously described examples.

**Figure 1.**
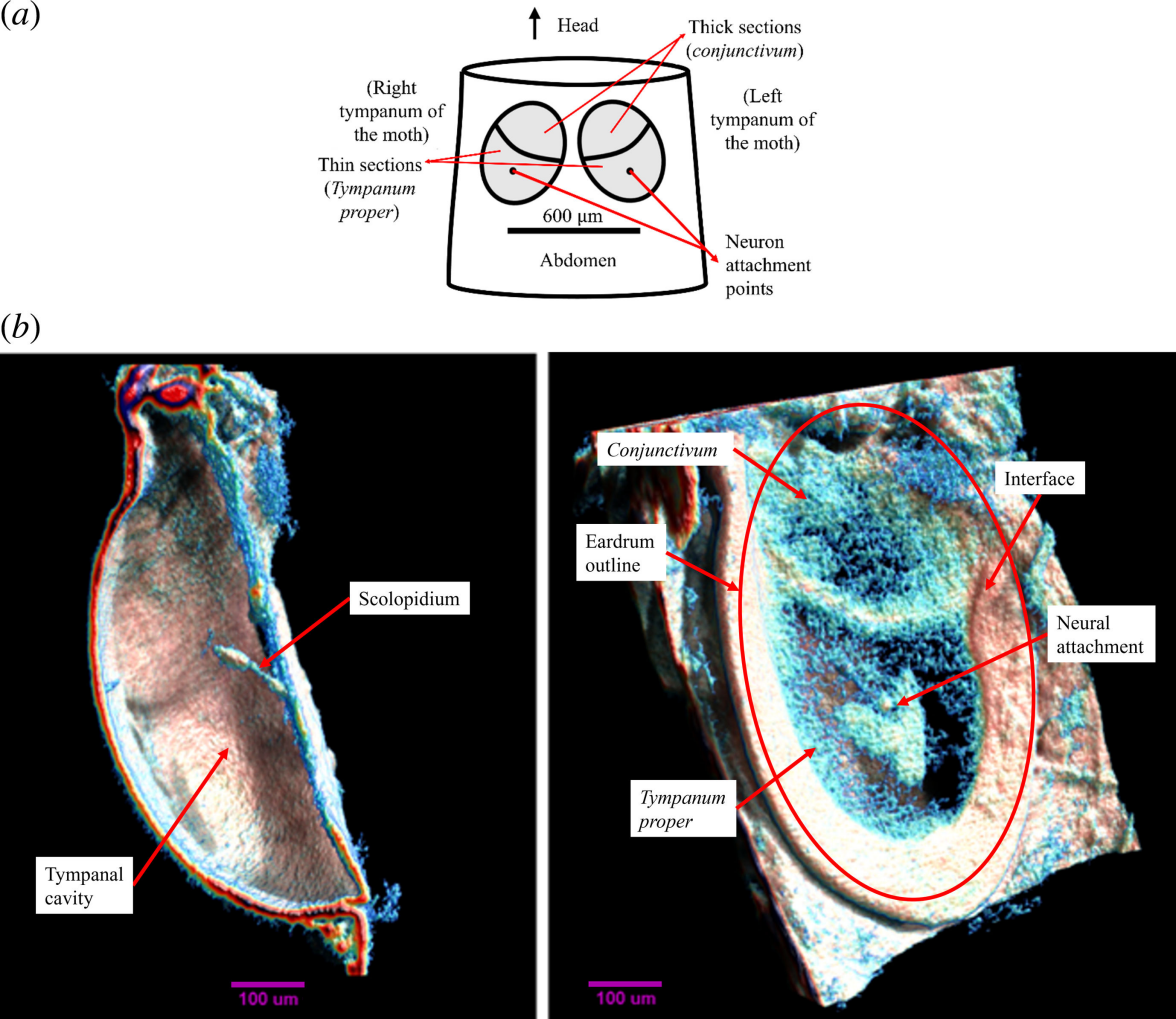
(*a*) Schematic representation of *Achroia grisella*’s abdomen, with the tympana, their sections, and the neural attachment points labelled (adapted from [27]). (*b*) X-ray microtomography voxel reconstruction of the eardrum of *A. grisella*. (Left) Lateral view cross-section along the major axis showing the tympanal cavity and the scolopidium. (Right) Front view of the tympanum showing the neuron attachment point and the border between upper and lower membranes [27].

*Achroia grisella* has two tympana located in its abdomen [26]. The eardrums are approximately elliptical in shape and have two distinct regions of different thicknesses; the thicker one is called *conjunctivum* and the thinner one is called *tympanum proper* (see figure 1*a*) [28]. A cluster of four auditory neurons directly attaches to the approximate centre of the thin section [29]. An X-ray microtomography voxel reconstruction of the eardrum of *A. grisella* is shown in figure 1*b*. No connection of any sort or tracheal system has been observed in X-ray scans of the moth, discarding mechanisms like those of *O. ochracea* or Orthoptera.

In addition, upon closer observation of the tracking process, the female moths are seen to zigzag in their approach to the males, which is inconsistent with binaural tracking [30]. On the other hand, experiments where one of the moth’s eardrums was pierced showed that the females were still able to approach a speaker reproducing the male singing, indicating monoaural directional hearing [31]. Due to the lack of known mechanisms that connect the tympana to each other or the outside and its apparent monoaural directional hearing, it is believed that *A. grisella* achieves directionality solely owing to the morphology of the eardrums themselves.

Seeking to understand biological systems, even when seemingly simple, requires the use of multiple tools and techniques. This includes X-rays for description of the tissues and structures, dissection, microscopy, etc. These are all mostly destructive or expensive, but one alternative where one can consider several of the factors governing the system is computer modelling and simulation. Modelling is used to simulate the system studied but also allows simplification of the features. Finite element modelling (FEM) is one type of computer simulation that can deal with complex systems. It has been used before to successfully model biological elements [32–34]. A model of the moth eardrum approximating the most salient features is therefore developed to observe the natural resonance modes and the directional response and is presented in the next sections.

## 2. Methods

The software COMSOL Multiphysics^®^ was used for the FEM. With FEM, the behaviour of a complex geometry with multiple governing equations is studied by reducing the geometry to a mesh of smaller finite elements. The intersection between elements, the nodes, is where the solutions are determined to satisfy the governing equations. In general terms, the finer the mesh (the smaller the elements that make it up), the better the results will be. Nevertheless, an important factor to consider is that there is a trade-off between computation accuracy and computational effort. Ideally, the mesh size for a determined geometry will be fine enough to achieve good results but not computationally cumbersome.

The structure simulating the eardrum is modelled using COMSOL’s Solid Acoustics module, specifically a shell interface, appropriate for thin plates with high bending stiffness. The dimensions and properties of the eardrum are taken from the existing literature. The average dimensions of the eardrum are taken from those reported by Knopek and Hintze-Podufal [29], and from unpublished work by Dr Andrew Reid (available as electronic supplementary material): major semi-axis of 335 µm, a minor semi-axis of 250 µm, *conjuctivum* thickness of 8 µm and *tympanum proper* of 3 µm. It is worth noting that there is no extensive literature regarding the particular tissue properties of *A. grisella*’s eardrum, so ranges of values for other types of insect cuticles are used instead. Young’s modulus values range from 1 MPa to over 20 GPa; volumetric mass density ranges from 1 to 1.3 g cm^−3^; Poisson’s ratio ranges between 0.02 and 0.5. The Poisson’s ratio is set as 0.35, and the volumetric mass density is chosen as 1180 g cm^−3^, both standard values for their ranges. Three values are tested for Young’s modulus, being the parameter with the widest range of values [35–37].

A circular uniform plate and elliptical uniform plate are first modelled, their eigenfrequencies being compared with the resolution of the mathematical equations for the same problems, serving as ground truth and first approximation to the model of the eardrum. Eigenfrequencies (also known as natural frequencies) are those at which a particular system is inclined to vibrate. The simulated results are found to match the mathematical ones well, but no directionality is expected or shown for the circular one, and very limited directionality is seen in the elliptical one, therefore discarding them as potential simplifications of the eardrum.

The shell interface is next modelled as an elliptical shape of major and minor semi-axes and two halves of different thicknesses, as indicated above. The interface separating the *tympanum proper* from the *conjunctivum* is displaced 15 µm along the major axis and parallel to the minor axis, making the *tympanum proper* slightly larger than the *conjunctivum*. Different values for Young’s modulus and the loaded mass are considered, as well as the position of the loaded mass in the *tympanum proper* [35].

The mass chosen is one of biological significance, equal to the total mass of the plate (obtained by using the volumetric mass density and the dimensions of the plate). In the moth, the outline is not perfectly elliptical, the boundary between sections is not a straight line, the change in thickness is a gradient and not a step, and the neural attachment point connects the tympanum to the back cavity of the eardrum. These simplifications in features are done while expecting the model to still account for the behaviour seen in the moth eardrum.

The first study ran for the shell interface is a preset eigenfrequency study to find the first six eigenfrequencies of the system. The mesh size is evaluated while running the eigenfrequency study for six different mesh sizes to determine which one to use going forward. A trend can be observed where the results improve less with the reduction in mesh size for the finer meshes. It is decided that COMSOL’s ‘Extremely fine’ mesh provides a good balance between the accuracy of the results and computational efficiency. The Mode Shape results node shows the deformation of the shell at the different eigenfrequencies.

COMSOL’s Pressure Acoustics module is then added to be used as a domain to account for the transmission of sound in air and therefore allow the study of directionality. A hemispherical domain is built around the shell, and air is assigned to it as material. A Multiphysics model is created for the boundaries between the shell domain and the air domain.

**Figure 2.**
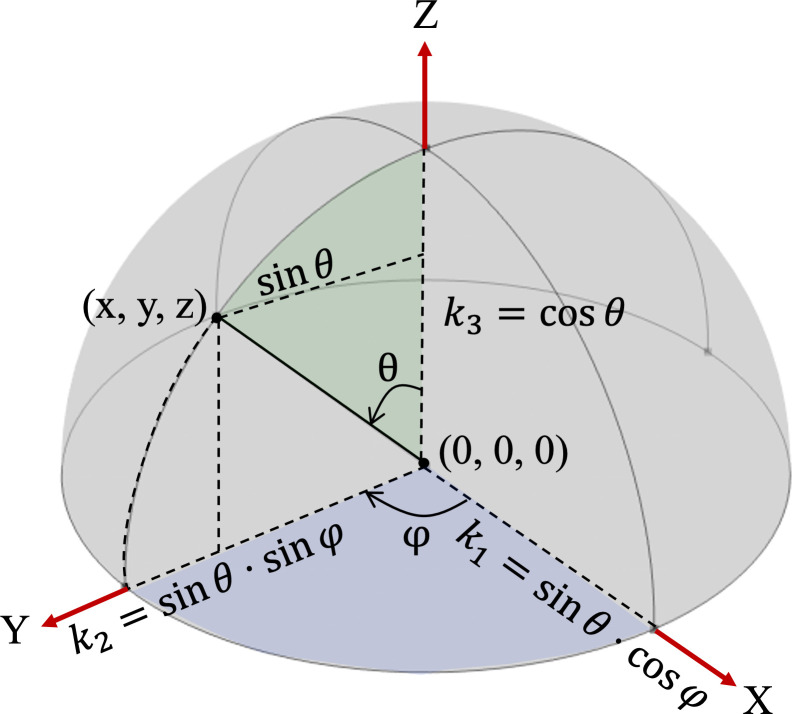
Representation of the elevation (*θ*) and azimuth (*φ*) angles and the vectors *k*_1_, *k*_2_ and *k*_3_.


A stimulus in the form of a spherical wave radiation of incident pressure field equal to 1 Pa is inserted. The stimulus is then rotated around the shell, where both azimuthal or polar (*φ*) and elevation (*θ*) angles can be controlled, and the direction of the provenance of the stimulus is determined by three vectors, *k*_1_, *k*_2_ and *k*_3_, dependent on the angles. These angles and vectors are defined in equation (2.1) and graphically represented in figure 2.

(2.1) $$\begin{array}{l} \left\{ \begin{array}{l} k_{1}=\sin\left(\theta \right)\cdot \cos\left(\varphi \right)\\ k_{2}=\sin\left(\theta \right)\cdot \sin\left(\varphi \right)\\ \;\;\;k_{3}=\cos\left(\theta \right) \end{array}\right. \end{array}$$

**Figure 3.**
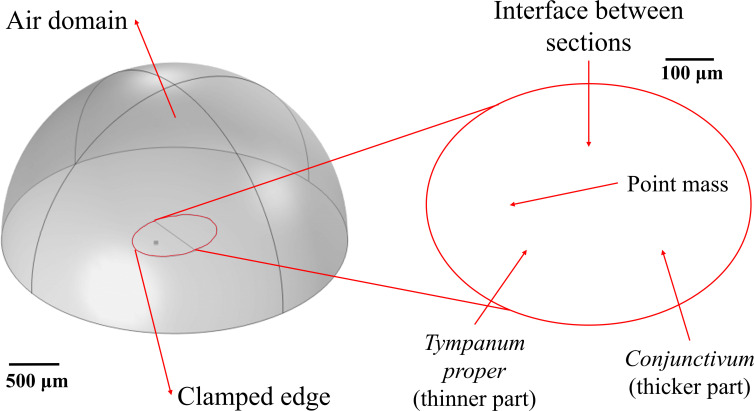
COMSOL geometry for the elliptical double-thickness plate with point mass.

A general frequency study is set with a sweep over the polar angle from 0° to 360° in intervals of 5°. The azimuthal angle can be changed but is maintained as fixed for each study. It is expected that the polar plot will not be a circle, which would represent omnidirectionality or equal response to sounds coming from all angles; instead, a directional response would be characterized by an asymmetric polar plot. Figure 3 shows the geometry described above as shown in the COMSOL software with a close-up of the shell.

## 3. Results

### 3.1. Eigenfrequencies, eigenmodes and parameter influence

**Figure 4.**
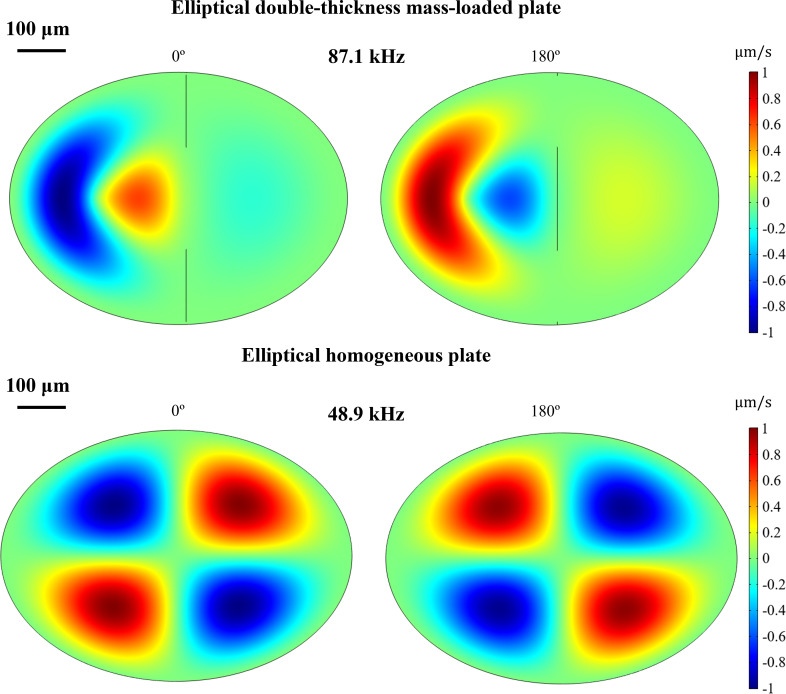
COMSOL simulation of the fifth eigenmode of an elliptical double-thickness mass-loaded plate (Young’s modulus 1 GPa, mass load of 1.556 µg, mass location 160 µm from interface into the *tympanum proper*) and the fifth eigenmode of an elliptical homogeneous plate (Young’s modulus 1 GPa, same dimensions). Two stills of opposite phases are shown to convey the maximum range of the eigenmode.

A preset eigenfrequency study is run in COMSOL for the first six eigenmodes for parameter values obtained from the literature (Young’s modulus 1 GPa, mass load of 1.556 µg, mass location 160 µm from interface into the *tympanum proper*), and the results returned show a fifth natural resonance most graphically alike to the displacement pattern described in the literature for the moth ear (figure 4). This consists of a maximum displacement peak close to the neural attachment point surrounded by a half-ring of lower amplitude and out of phase with the main peak, with a broader bump on the thicker section of the eardrum, also of approximate phase to the main peak [30,38]. The inclusion of the two-thickness feature and the point mass improve the resemblance to the moth eardrum pattern greatly in comparison with the homogeneous elliptical plate (see comparison in figure 4).

The simulation was run with three values for Young’s modulus, which is the parameter with the widest range of values in the literature for insect cuticles (see table 1). Both ends of the spectrum were used (1 MPa and 20 GPa), and then a third value of 1 GPa was chosen because it is somewhat in the middle of the spectrum for sclerotized cuticles [35] and because it produces a fifth resonant mode at a frequency close to the male’s mating call and the female moth’s maximum sensitivity. The moth ear’s maximum sensitivity was found to be 90 kHz through laser vibrometry of the eardrum exposed to artificial signals built to emulate the male’s call [39]. The effect on the Young’s modulus on the system’s behaviour, while keeping the other two parameters constant, is impactful, which is to be expected considering the wide range between values. For the fifth frequency, the values change from 2.9 to 87.1 to 411.7 kHz for 1 MPa, 1 GPa and 20 GPa respectively.

**Table 1.** Simulated eigenfrequencies for the elliptical double-thickness plate clamped all around. Three different values for Young’s modulus (mass load of 1.556 µg, mass location 160 µm from the interface into the *tympanum proper*).

Young’s modulus			
eigenfrequency	1 **MPa**	1 **GPa**	20 **GPa**
first	228.5 Hz	7.0 kHz	32.3 kHz
second	1.2 kHz	38.1 kHz	165.5 kHz
third	1.9 kHz	58.8 kHz	263.2 kHz
fourth	2.1 kHz	70.0 kHz	297.4 kHz
fifth	2.9 kHz	87.1 kHz	411.7 kHz
sixth	3.0 kHz	92.1 kHz	481.4 kHz

Next, the location of the loaded mass point is modified while keeping the other two parameters constant and it is seen to greatly affect the fourth, fifth and sixth resonant frequencies, eventually exchanging the positions of the fifth and sixth (see table 2). The mode shapes are even more affected with the displacement of the mass point as little as 15 µm closer to or further from the interface between *tympanum proper* and *conjunctivum*. For a location of the neural attachment point mass of 170.95 µm into the tympanum, the fifth and sixth modes become so close as to essentially degenerate at 92.07 and 92.08 kHz.

**Table 2.** Simulated eigenfrequencies for the elliptical double-thickness plate clamped all around. Three different values for the location of the mass point (Young’s modulus 1 GPa, mass load of 1.556 µg).

point mass location						
eigenfrequency	175 **μm**		160 **μm**		145 **μm**	
first	7.5 kHz		7.0 kHz		6.9 kHz	
second	37.0 kHz		38.1 kHz		39.3 kHz	
third	58.9 kHz		58.8 kHz		58.8 kHz	
fourth	66.5 kHz	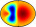	70.0 kHz	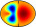	72.4 kHz	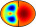
fifth	92.1 kHz	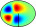	87.1 kHz	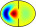	80.2 kHz	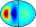
sixth	93.6 kHz	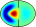	92.1 kHz	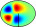	92.1 kHz	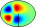

Contrarily, the mass itself doubling or halving (while keeping the other two parameters constant) does not affect the eigenfrequencies or eigenmodes notably (see table 3). If the mass is removed completely, the resonant frequencies increase significantly, and the fifth and sixth modes are again switched, with the sixth mode in particular changing shape considerably. The system is thus shown to be sensitive to the variation of multiple parameters, but more so to be dependent on Young’s modulus and mass position.

**Table 3.** Simulated eigenfrequencies for the elliptical double-thickness plate clamped all around. Four different values for the point mass accounting for the neural attachment point, including its complete absence (Young’s modulus 1 GPa, mass location 160 µm from interface into the *tympanum proper*).

point mass				
eigenfrequency	0.778 **μg**	1.556 **μg**	3.113 **μg**	no mass
first	9.7 kHz	7.0 kHz	5.02 kHz	29.2 kHz
second	38.3 kHz	38.1 kHz	38.0 kHz	46.4 kHz
third	58.8 kHz	58.8 kHz	58.8 kHz	58.8 kHz
fourth	70.4 kHz	70.0 kHz	69.8 kHz	78.6 kHz
fifth	87.3 kHz	87.1 kHz	86.9 kHz	92.1 kHz
sixth	92.1 kHz	92.1 kHz	92.1 kHz	99.0 kHz

### 3.2. Directivity analysis

**Figure 5.**
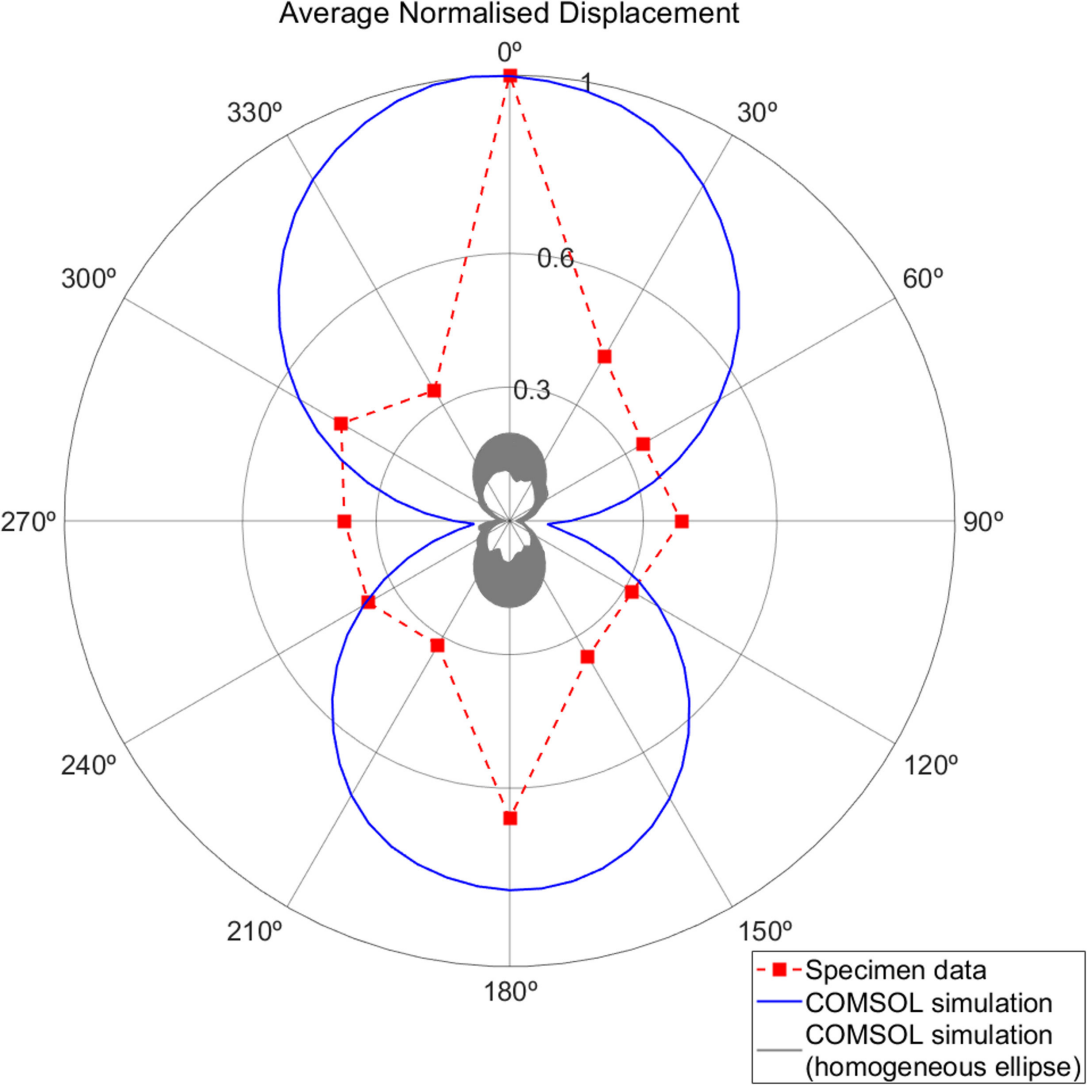
Comparison in a polar plot of the data points obtained from a real specimen by Dr Andrew Reid and the COMSOL simulation (87.1 kHz, Young’s modulus 1 GPa, mass load of 1.556 µg, mass location 160 µm from interface into the tympanum), where both have been normalized. The grey-shaded area represents the normalized directionality response of a homogeneous elliptical plate (no thickness change and no mass load) for three different thicknesses. *Achroia grisella* data is taken from figure 4*a* from Reid *et al*. [30]

The directivity analysis requires the addition of the Pressure Acoustics module, as described in the §2. The eigenfrequency study is run again to ensure that the results remain consistent, which is true as long as the mesh element sizes are kept constant. The results of displacement measured at the precise neural attachment point (location of the point mass) while sweeping over the azimuthal angle are normalized with respect to the maximum and presented in figure 5 in comparison with data obtained from a real moth. For the real moth measurements, the specimen is prepared by removing the ventral cleft and legs and the body being turned upside down, so the tympanum is perpendicular to the scanning laser vibrometer used to measure tympanal displacement at the neural attachment point. The Pearson correlation coefficient (PCC) for the two datasets is 0.482 and the *p*-value is 0.0957. For the PCC, values above 0.5 are considered strongly correlated; for the *p*‐value, a commonly chosen level of significance for small sample sizes is 0.1 [40]. The PCC is just under the standard accepted threshold for strong correlation, and the *p*-value is within significance considering the nature of the research and the characteristics of the datasets.

Figure 5 also shows a grey-shaded section that corresponds to the range of normalized directionality responses of a homogeneous elliptical plate (no thickness change and no mass load) for three different thicknesses: that of the thin section, that of the thick section and the average of the two. The increase in thickness makes the eigenfrequencies higher, as expected, and some artefacts due to mesh size at higher frequencies can be seen. Nevertheless, the maximum displacement is approximately 10 times less than that of the full model.

The simulation, as well as the real moth data, show that maximum displacement is seen when sound comes from the 0° direction, which corresponds to the front of the moth, a decrease in the response when sound comes from the left and right sides (90° and 270°), and a slight increase when sound comes from the back (180°) but less than from the front (0°). It must be noted that there is a disparity between the sampling of the simulated model and the real moth data, where the data captured for the specimen is sampled more sparsely. Nevertheless, the main features of both sets of data are in agreement.

### 3.3. Damping modelling

The results presented so far are under the assumption of no damping in the system, which means that motion would continue uninterrupted in the absence of external forces. But, in reality, damping is present and accounts for the transfer of energy that causes the decay of the waves that travel through the system. When damping is present, maximum displacement and equilibrium positions in a particular eigenmode are not synchronized in all parts of the system, but instead, the peak values for maxima and equilibrium positions are not reached at the same time and the imaginary part of the eigenfrequency reflects the phase information. This more accurately represents what is seen in the moth eardrum [30,38].

Damping in COMSOL can be modelled in various ways, e.g. loss factor damping, Rayleigh damping, viscous damping, modal damping or thermoelastic damping, among others. Nonetheless, damping can be difficult to determine in a specific system. If considering, for example, loss factor damping, a very small change in loss factor can impact results greatly. Viscous damping modelling is favoured due to a simple mathematical expression. Its inclusion in the model presented here is an attempt to qualitatively improve the similarity of the model to the real moth eardrum. The addition of viscous damping to the model, through the consideration of air as a viscous gas, produces the appearance of complex eigenfrequencies, where the imaginary part corresponds to the decaying part of the solution.

**Figure 6.**
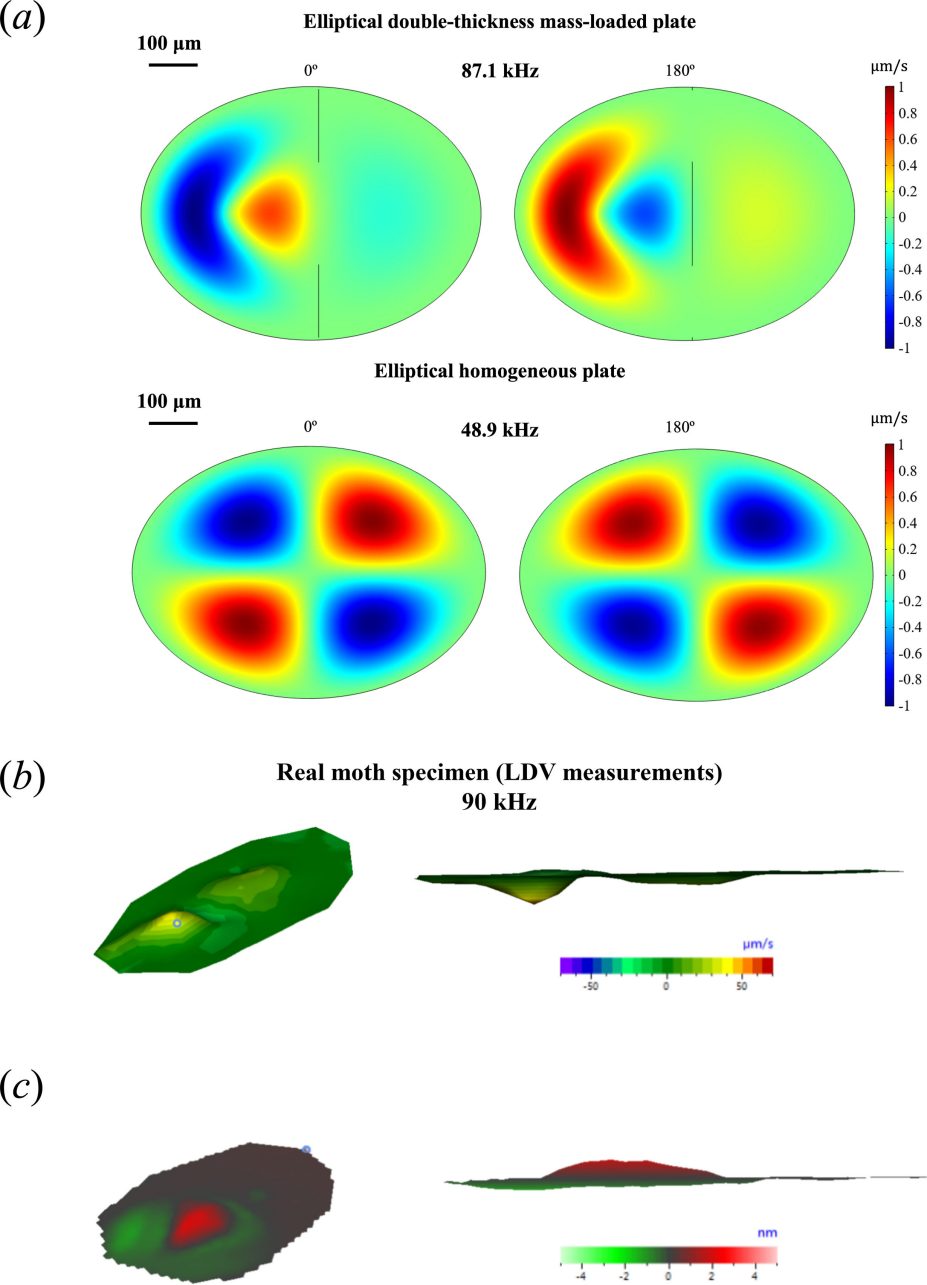
(*a*) Eigenmode for the eigenfrequency found at 76.145+34.941 *i* kHz for an elliptical double-thickness plate with a mass-loaded point and damping induced by a viscous model for air. Two stills of different phases are shown to convey the range of the eigenmode, and XY and XZ views are provided to evidence the peaks and the phase relation between them. (*b*) Laser Doppler velocimetry (LDV) measurement taken from a moth specimen showing the velocity at which the tympanum surface moves. (*c*) LDV measurement taken from a moth specimen showing displacement of the tympanum surface. (*b*) and (*c*) were originally published in [27].

The new displacement pattern of the fifth eigenfrequency (see figure 6*a*) consists of a peak with maximum displacement near the point loaded with mass, surrounded by a series of secondary peaks, out of phase but not in exact opposition of phase, and a broader bump in the thick region, of a similar phase to that of the peak ring. For the considered model, the addition of viscous damping increases the apparent likeness to the vibration pattern on the moth ear (figure 6*b*,*c*).

## 4. Discussion and conclusions

This research presents the first work to model the eardrum of the moth *A. grisella* to explain its monoaural directional hearing. FEM proves a useful tool to unravel the inner workings of *A. grisella*’s oddly simple directional ear. The results presented show that a simplification from the complex natural morphology of *A. grisella*’s ear to an elliptical double-thickness mass-loaded plate bears similarities to the eardrum’s movements. A mode shape (the fifth eigenmode at 87.1 kHz) is identified for one model (centred neural attachment point at 160 µm, mass to account for the neural attachment of the same mass as that of the whole plate of 1.556 µg, Young’s modulus of 1 GPa) that resembles the displacement of the eardrum under exposure to a signal of approximately 90 kHz (that of the male mating signal).

Furthermore, displacement at the neural attachment point for that natural resonance mode (which resembles the displacement of the moth tympanum) presents a directional polar pattern with maximum response to sounds coming from the front of the moth, therefore indicating that the morphology of the system is conferring it with directionality. The moth eardrums sit at an angle of 30° with respect to the midline of the moth body (figure 1*a*). This means that the 0° direction of the major axis of the moth eardrum corresponds to either −30° or 30° with respect to the moth’s midline (left or right ear respectively), which is consistent with the fact that the moth is seen to zigzag while tracking the male calls. The PCC and the *p*-value for the two datasets (simulated model and moth specimen) are calculated, and the values support the correlation of the two polar plots. Considering that the model is based on limited features of the moth eardrum, these results are promising and would probably be improved if more features were incorporated into simulation. Lastly, viscous damping is added to the system through the gas model for the air domain, which alters the eigenfrequencies, making them complex-valued, and increases the qualitative likeness to the observed motion of the actual moth tympana.

Future work for the model includes the modelling of the body around the eardrum and the ventral cleft instead of the consideration of the eardrum in isolation. The body could be approximated by a simple volume like a cylinder, or a three-dimensional scan of an actual moth could be imported and used in COMSOL. Additionally, the somewhat upright natural walking position of the female *A. grisella* most frequently adopted when tracking the male should also be taken into account, since wavefronts will then not be perfectly parallel or perpendicular to the eardrum plane. Precise values for the characteristics of insect cuticle would also be an improvement to the model since the models used are wide-ranging and approximated from the literature, but not specific for *A. grisella*.

It is worth noting that thickness-graded eardrums are common in many insects, but the characteristic has not always been found to relate to directionality. *Galleria mellonella* (Fabricius, 1798) (Lepidoptera: Pyralidae), also known as the greater wax moth, presents a similar eardrum to *A. grisella* but shows no directional response, and acoustic calling plays no part in its mating process [30]. Thickness gradation in locusts, *Schistocerca gregaria* (Forsskål, 1775) (Orthoptera: Acrididae), has been related to tonotopy but not directionality [41]. Katydids (Orthoptera: Tettigoniidae) eardrum thickness variation is limited and seemingly unrelated to directional hearing [42]. It is possible that thickness gradation in the eardrum of these insects plays a minor role in directionality, but other systems are present and more efficient (such as multiple pathways in Orthoptera), or directional hearing not necessary and therefore not exploited (such as is the case for *G. mellonella*).

Overall, the research presented by the authors helps solve the mystery of the monoaural directional hearing of the moth *A. grisella*. Directional hearing at such a small scale could be of inspiration to other small-sized applications that are challenging in the current state of the art, like microphones for smartphones or hearing aids, where the regular microphones find problems exclusive to the reduced scale. The problem of hearing in the moth is explored by leveraging knowledge on biology, physics and engineering, and exploiting FEM, given that it sits at the intersection of the three fields.

## Data Availability

The data used for morphology measurements of the moth eardrum is publicly available at [43].
